# Supplementary material for: A socio-ecological approach to understanding the factors influencing the uptake of intermittent preventive treatment of malaria in pregnancy (IPTp) in South-Western Nigeria
Source: PLoS One. 2021 Mar 15;16(3):e0248412. doi: 10.1371/journal.pone.0248412 (PMC7959387; doi:10.1371/journal.pone.0248412)
Supplement: S1 File — (DOCX) [file pone.0248412.s001.docx]

**S1 File_ COREQ (Consolidated criteria for Reporting Qualitative research) Checklist**

A checklist of items that should be included in reports of qualitative research. You must report the page number in your manuscript where you consider each of the items listed in this checklist. If you have not included this information, either revise your manuscript accordingly before submitting or note N/A.

| **No.** | **Topic** | **Guide Questions/Description** | **Page No.** |
| --- | --- | --- | --- |
| **Domain 1: RESEARCH TEAM AND REFLEXIVITY** | | | |
| Personal characteristics | | | |
| 1 | Interviewer/facilitator | 14 RAs conducted all interviews and discussions | 4 |
| 2 | Credentials | GNN(PhD Global Health), AO (MBChB, MPH, FMCOG, FWACS), MOO (PhD Demography and Social Statistics), OW (MBBS, Dr. Med. Sci, FMCP, FWACP), DA (MBBS, MD, FRCOG, LLM ) | 14 |
| 3 | Occupation | GNN (Social Scientist), AO (Senior Lecturer and Consultant Obstetrician/Gynaecologist), MO (Demographer and Social Statistician), OW (Professor of Clinical Pharmacology), DA (Professor of Obstetrics and Gynaecology) | 14 |
| 4 | Gender | Three researchers are females and two are males | NA |
| 5 | Experience and training | GNN, AO and MO have extensive experience in conducting qualitative research studies. OW and DA have some experience conducting and supervising qualitative research | NA |
| Relationship with participants | | | |
| 6 | Relationship established | No | 4 |
| 7 | Participant knowledge of the interviewers | No |  |
| 8 | Interviewer characteristics | Yes | 4 |
| **DOMAIN 2: STUDY DESIGN** | | | |
| Theoretical framework | | | |
| 9 | Methodological orientation and Theory | Grounded theory | 4 |
| Participant selection | | |  |
| 10 | Sampling | Purposive | 4 |
| 11 | Method of approach | Pregnant women were identified and recruited with at ANC facilities. They facilitated interviews with their caregivers and community leaders. Public healthcare providers, TBAs and faith-based birth attendants were purposively selected | 4 |
| 12 | Sample size | 201 | 4 |
| 13 | Non-participation | No identified participant declined participation | 4 |
| Setting | | | |
| 14 | Setting of data collection | Homes and community meeting places | 4 |
| 15 | Presence of non-participants | No | 4 |
| 16 | Description of sample | Table 1. Characteristics of study participants | 5 |
| Data collection | | | |
| 17 | Interview guide | We used an interview guide to guide the discussion | 4 |
| 18 | Repeat interviews | No | 4 |
| 19 | Audio/visual recording | All interviews were digitally recorded | 4 |
| 20 | Field notes | No | 4 |
| 21 | Duration | 30-65minutes | 4 |
| 22 | Data saturation | Yes | 4 |
| 23 | Transcripts returned | No | 4 |
| **DOMAIN 3: ANALYSIS AND FINDINGS** | | | |
| Data analysis | | | |
| 24 | Number of data coders | 3 | 4 |
| 25 | Description of the coding tree | Yes | 4 |
| 26 | Derivation of themes | Themes were pre-identified and evolved from the data | 4 |
| 27 | Software | Nvivo version 11.0 Pro | 4 |
| 28 | Participant checking | No | NA |
| Reporting | | | |
| 29 | Quotations presented | Yes | 6-11 |
| 30 | Data and findings consistent | Yes | 10-13 |
| 31 | Clarity of major themes | Yes | 6&8 |
| 32 | Clarity of minor themes | Yes | 6-10 |
