# Supplementary material for: A socio-ecological approach to understanding the factors influencing the uptake of intermittent preventive treatment of malaria in pregnancy (IPTp) in South-Western Nigeria
Source: PLoS One. 2021 Mar 15;16(3):e0248412. doi: 10.1371/journal.pone.0248412 (PMC7959387; doi:10.1371/journal.pone.0248412)
Supplement: S2 File — (PDF) [file pone.0248412.s002.pdf]

MIP Study: Interview guides (Qualitative)

Aim: to explore views and experiences regarding the implementation of WHO and national malaria prevention and treatment guidelines.

Topics will include experiences and perceptions about ANC utilization, ITN use, IPTp compliance and treatment-seeking for MIP.

<u>FGD guide for pregnant women</u>

- Introduction
- Icebreaker – How has your pregnancy experience been so far?

Pregnancy care practices

- What are some of the things a woman should do once she is pregnant [probe for local practices/dos and don’ts surrounding pregnancy, antenatal or TBA attendance]
- Why do we attend antenatal or require the services of a TBA? [probe for perceptions and experiences at antenatal and using TBAs]

Malaria prevention and treatment in pregnancy

- Do pregnant women get malaria? if yes, what are the things we do to prevent ourselves from getting malaria? [probe for local and antenatal practices] If antenatal is mentioned, probe for ITN use, why, what services and drugs are given and probe for IPTp, perceptions regarding IPTp, knowledge of IPTp, expected frequency of intake versus actual frequency of intake, how IPTp is given, perceived side effects, reasons to take and reasons to not take IPTp
- In our condition, when we get malaria, what happens? [probe for experiences, symptoms, treatment options- if local, what ingredients, if biomedical, what drugs]
- What is your view on using drugs to prevent MIP? How often do you take IPT in this pregnancy?
- Are there ways of preventing or treating MIP in this community? What is done? [*Probe – whom do you report to? How? Where? When?*]

Malaria effects/complications in pregnancy

- What can happen to us and our babies if we get malaria during our pregnancy? [probe for miscarriages, stillbirths, premature delivery, LBW]

Facilitators and/barriers to malaria prevention and treatment

- Have we experienced malaria during our pregnancies? How did we treat it? [probe for difficulties]
- What has made (or would make) the prevention or treatment of MIP easier for you? [*Prompt – examples*]
- Where do you intend to deliver your baby? How did you decide on where to deliver your baby (past and index pregnancies)?

Is there anything you would like to add?

MIP Study: Interview guides (Qualitative)

Aim: to explore views and experiences regarding the implementation of WHO and national malaria prevention and treatment guidelines.

<u>IDI guide for Pregnant Women</u>

As explained earlier, we want to hear your views and experiences regarding pregnancy and healthcare seeking for MIP. I will be asking you to share your views and experiences regarding antenatal care, the quality of the services you receive there, malaria, its prevention and treatment during pregnancy and the reasons for your choices. I would also ask you to share the experiences of other pregnant women who had malaria that you know of in this your community. I will not interrupt but may ask you to stop in between to clarify some of your answers by probing further on what you told us and what we want to know more about, so please be free with us and talk. Again, whatever we discuss here is confidential and we will not use any information that might identify you in our reports. Your views are very important as they will contribute to improve healthcare for pregnancy and malaria in pregnancy. Please take your time and let me know when we can start the interview.

Note for Interviewer: Do not ask direct questions but rather prompt by – give us an example /could you please tell me more?

Q: -- When did you start to use the maternity service in this pregnancy (Probe to know if there was an earlier pregnancy and repeat same question for the earlier pregnancy? What do you think about the maternity services available in this community (Probe further on the opinion positive or negative?

- Kindly, tell me about where you visit for maternity services in this pregnancy?

  --Why this facility or Home?

  --What services are provided?

  [*if medicines are mentioned, probe for what illnesses, probe for IPTp here, reasons for taking or not taking, perceptions, frequency, side effects and general knowledge on IPTp*].

  --What do you think about the services provided to you at this facility / Home

  [*probe for quality and discrimination and why, probe for comfortability*]

  -- Would you prefer another place to obtain maternity services? [*Probe for the name, location and why*]

  -What do you think about how pregnant women are treated where you receive maternity care?

  [*Probe to know if treated equally and why she thinks so*]

  --Apart from this facility/Home, have you ever been elsewhere for maternity service? (*Probe further*)

- Please tell me about how you access services at your maternity care center / Home?

  --Since you registered for this maternity service, have you ever skipped a visit? When? Why?

  -- Who pays when you use maternity services?

  --How convenient is it for you or this person to pay?

  -- How affordable are these services for you?

  --Do you have to inform anyone before going to or receiving maternity services? (If Yes, probe to know who and why, if no, why)

  --how do you choose where you go for maternity services?

Now, I want to ask you to share your illness and treatment experience

- Have you ever been sick in this pregnancy? (if yes, probe to know what happened; if no, probe to know the steps she has been taking to remain healthy in her current state)
  --did you seek treatment for the illness?
  --what type of treatment?
  --where did you seek treatment?
  --why the choice of treatment?
  --who influenced your decision for the place of treatment and choice of treatment?
  --who paid for the treatment?

Have you ever had malaria during pregnancy? If yes, kindly share your experience with me. [if no, ask of a friend or relative's experience]

- Kindly share with me how malaria is handled during pregnancy in this community (*Probe to know what the local name is– practices and norms for prevention and treatment, consequence etc*)
  --what is your source of information on malaria in pregnancy?
  --what are the things you have done to prevent malaria during this pregnancy? [*Prompt –measures taken? local remedies, self-medication (names of drugs), IPT (how many times), ITN use, antenatal attendance*]
  --How do pregnant women in this community treat malaria when they have malaria?
  -- the last time you (or another pregnant woman you know) had malaria, did you (or she) seek treatment?
  --where did you seek treatment?
  --why the choice of treatment?
  ---how did you decide on choice and where to seek treatment?
  --who pays for the treatment?

  Is there anything else you would like to tell me?

MIP Study: Interview guides (Qualitative)

Aim: to explore views and experiences regarding the implementation of WHO and national malaria prevention and treatment guidelines.

IDI Guide for Mother-in-Law / Grandmother / Spouse/Partner

- Could you tell me the things a woman should do once she realizes that she is pregnant? [probe for dos and don'ts]
  -- How can a pregnant woman ensure she is healthy or fit all through her pregnancy? (Probe every response given, ask for local names etc)
  ---- Could you share with me how successfully took your own pregnancy till delivery?
  ---- where there any things you heard or where told to do or not to do during your pregnancy (probe for hearsays, sources of hearsay etc.)

- What do you think about women attending maternity service during pregnancy? (Probe for why)
  --When should a woman seek maternity services during pregnancy? (why?)
  --Where should a woman seek maternity service during pregnancy (Probe to know why?)
  -- what do you think about the way that maternity services are delivered in this community? (probe for the timing, privacy, cost, distance, attitude of healthcare workers, interval between visits, waiting time etc.)
  --- could you share any of your personal experiences or observations (good and or bad) you have had with the maternity service center in your area.
  --**For older women -** how are you supporting your daughter/daughter-in-law/granddaughter in her pregnancy? (probe for choice of maternity service and why, timely registration and attendance for antenatal care visits, adherence with prescribed medications etc).
  – **For spouse/partner -** how do you support your wife/partner in her pregnancy? (probe for choice of maternity service and why, timely registration and attendance for antenatal care visits, adherence with prescribed medications etc).

- What common ailments affect pregnant women in this community? (Probe for names including local names, ask for personal experiences or of others known to her/him)
  --how are they diagnosed and treated (Probe for the diagnosis and treatment for each ailment)

- Please tell us the diagnosis and treatment of malaria for a pregnant woman (Probe for more information)
  -- **For older women -** how do you prevent or support your daughter/ daughter-in-law/granddaughter from having malaria during her pregnancy?
  -- **For older women -** has your daughter-in-law/ granddaughter/ daughter ever had malaria in this pregnancy? (what happened)
  -- **For spouse/partner -** how do you prevent or support your wife/partner from having malaria during her pregnancy?
  -- **For spouse/partner -** has your wife/partner ever had malaria in this pregnancy? (what happened)
  --How was it treated? (probe for treatment options, reasons for choice, preferred treatment option, role in choice of treatment option)
  --how did you help her decide on the treatment option, where and when she got treatment?
  --- what do you think would have happened to her and/or her baby if she did not get treated for malaria?
  --How can you support her in getting treatment for malaria during pregnancy in time? (probe for the definition of the timely treatment, probe for adherence to malaria treatment)

---how can you support her to have a healthy pregnancy and delivery? (probe for steps taken and timing, probe for experiences that shows the support)

MIP Study: Interview guides (Qualitative)

Aim: to explore views and experiences regarding the implementation of WHO and national malaria prevention and treatment guidelines.

Topics will include experiences and perceptions about ANC utilization, ITN use, IPTp compliance and treatment-seeking for MIP.

<u>Interview guide for Traditional birth attendants (TBA), Community health workers (CHW) and HCPs</u>

Introduction

- Icebreaker: could you share your experiences on how you came to be TBAs/CHWs/HCPs?
- What services do you provide? [*Probe – which ANC services do you provide?*]
- What do you think about antenatal care?
- How can a pregnant woman prevent malaria during her pregnancy? How do you support her to prevent malaria during pregnancy? [*Prompt – which measures do you provide? Are you aware of any national guidelines (government recommendations) for preventing MIP?*]
- What do you think about using medicines to prevent malaria during pregnancy? [*Probe for what medicines? IPTp – examples*]
- How do you treat pregnant women who fall sick with malaria? [*Probe – how is it diagnosed? What do you do? types of local remedies and or medicines given*]
- What will happen to a pregnant woman and/or her baby when she has malaria? (probe for if untreated what happens, miscarriages, stillbirths, LBW, premature births etc)
- Can you share any experiences where a pregnant woman has had malaria and how it affected her pregnancy, and her baby?
- Have you experienced any difficulties when treating malaria in the pregnant women that come to you? What happened? How did you handle it? [*Prompt - examples*]
- How can you be supported to help prevent or treat malaria in pregnant women? [*Prompt examples*]
- Is there anything you would like to add?

MIP Study: Interview guides (Qualitative)

Aim: to explore views and experiences regarding the implementation of WHO and national malaria prevention and treatment guidelines.

Topics will include experiences and perceptions about ANC utilization, ITN use, IPTp compliance and treatment-seeking for MIP.

<u>Interview guide for community leaders</u>

- Introduction
- Icebreaker: please tell me about your responsibilities as a leader in this community?
- what is your view about the kind of care available for pregnant women in your community? [*Probe – what type of care is available and perception regarding each type of care*]
- What do you think about antenatal care? What is your view about the maternity care services available in the community? What do you think about their costs?
- In your opinion, how do community members perceive these services? (probe for male and female views)
- How do pregnant women decide on when, where and which maternity services to use during pregnancy?
- How do we prevent malaria during pregnancy in our community here? What is done and how do we know which measures to use? (probe for ITN use, local remedies, antenatal attendance)
- How do we treat malaria illness in pregnant women? What is done? [*Probe – by whom? how? where?*]
- Could you share some experiences regarding the difficulties with malaria prevention or treatment in pregnant women? [*Prompt - examples*]
- How can we improve prevention or treatment of MIP in our community? [*Prompt – examples*]
- Is there anything you would like to add?
