# Supplementary material for: A socio-ecological approach to understanding the factors influencing the uptake of intermittent preventive treatment of malaria in pregnancy (IPTp) in South-Western Nigeria
Source: PLoS One. 2021 Mar 15;16(3):e0248412. doi: 10.1371/journal.pone.0248412 (PMC7959387; doi:10.1371/journal.pone.0248412)
Supplement: Attachment — Submitted filename: Response to reviewers comments.docx [file pone.0248412.s004.docx]

| **A socio-ecological approach to understanding the factors influencing the uptake of intermittent preventive treatment of malaria in pregnancy (IPTp) in South Western Nigeria _R1** | |
| --- | --- |
| **We thank the reviewers for making time to review our manuscript and making insightful comments which have contributed immensely in further shaping our manuscript. Below, we provide a point by the point-specific response to the comments of our reviewers.** | |
| Specific reviewer comments | Authors Response |
| Editorial team | |
| 1. Please ensure that your manuscript meets PLOS ONE's style requirements | Revised as suggested throughout the manuscript. |
| 2. Please remove any funding-related text from the manuscript and let us know how you would like to update your Funding Statement | We have removed funding-related text from page 25 as requested. We are satisfied with the funding statement and do not need to update it. |
| 3. In your revised cover letter, please address the following prompts: If there are ethical or legal restrictions on sharing a de-identified data set, please explain them in detail (e.g., data contain potentially identifying or sensitive patient information) and who has imposed them (e.g., an ethics committee). Please also provide contact information for a data access committee, ethics committee, or other institutional body to which data requests may be sent | Due to potentially identifying or sensitive patient information, there are ethical restrictions on sharing de-identified data. The data is available from the Ethics Review Board of Babcock University (contact via [olaleyea@babcock.edu.ng](mailto:olaleyea@babcock.edu.ng)) for researchers who meet the criteria for access to confidential data. |
| 4. Please ensure that you have an ORCID iD and that it is validated in Editorial Manager. To do this, go to ‘Update my Information’ (in the upper left-hand corner of the main menu), and click on the Fetch/Validate link next to the ORCID field. This will take you to the ORCID site and allow you to create a new iD or authenticate a pre-existing iD in Editorial Manager. | The corresponding author has tried to update her ORCID ID in the editorial manager but this hasn’t been possible because of the text “ *an identical, validated ORCID already exists in the database. An ORCID may be linked to only one record in the database and so you may have registered another user record in the past and retrieved/validated your ORCID for use with that.* “My ORCID ID is <https://orcid.org/0000-0002-1113-3832>. |
| Reviewer #1: | |
| 1. Line 23. “This study explores…” it should be with past tense – “explored”. | Revised on Page 2 line 23 |
| 2. In the last sentence, two words are duplicating “improving… xxx …..improving….xxx”. Please rewrite. | Revised on Page 3 line 43 |
| 3. Line 47-48: please update the reference. The WHO world malaria report 2019 is available for updated data. | Updated as suggested on Page 4 lines 46-48 |
| 4. Line 64-67: the sentence is not clear in writing. Please rewrite. | Revised as suggested on Page 4 lines 64-66 |
| 5. Objectives of the study is missing in the introduction. Please add the objectives at the end of the introduction. | This is already stated on Page 5, lines 81- 84 |
| 6. Methods: is the sample size for SSI and FGD were pre-defined or how the samples size was determined? Did you continue the interviews until the information (or data) was saturated? Please add. | Revised as suggested on pages 7-8, lines 134-137 |
| 7. Data analysis: please mention data analysis method (eg. “thematic approach” as stated in the abstract.) | Included as suggested on Page 8, lines 153-154 |
| 8. Table 1: A. FGD participants is suggested to change as followed. | We have revised the table as suggested with additional details on Pages 9-10 |
| 9. “ID” column is suggested to delete from both table A and B. Please delete “duration” column from table B. | We have removed “duration” as suggested. We have also changed ID to quotation ID on Page 9 because we would prefer not to describe the characteristics for every quote. In particular, the FGD quotations are composed of statements from between 6 to 10 participants, which makes it impossible to attribute and describe each quote. |
| 10. The number of quotations must be reduced. Recommend to select ONE best suitable quotation that reflect to the finding description is good enough (a maximum of two, not more than two). | We have significantly reduced our quotations on Pages 11- 19. However, the study uses a socio-ecological framework and as such for each level and for the various sources, we have tried to include a relevant quotation to provide evidence for our results. |
| 11. Participant’s ID number [eg. FGD-PW-TBA-S-02] must be removed from the selected quotation. Instead, please describe [eg. a 28-year pregnant woman from FGD who took ANC at TBA clinic (OR) a pregnant woman from FGD who took ANC at TBA clinic] | This suggestion would be ideal for one on one interviews but would not suit our FGD quotations. The quotations from the FGDs do not come from one participant but is a collection of statements from several participants in each FGD in response to a question. As is common in presenting qualitative data and FGD quotations, in particular, we use “…” to show breaks in statements from multiple participants. Secondly, this is why we have the quotation description above so that we can minimise the use of descriptions for each quotation which would only increase our word count. |
| 12. Some quotations are related to mis-believe, please describe about it in the description above, what kind of mis-believe they have. (eg. line 150-156) | We have elaborated on this on Page 11, lines 169-172 |
| 13. Line 145-146: please mention what are the “personal and social network experiences”. | Revised and explained as suggested on Page 11 lines 172-175 |
| 14. Line 163: the descriptions are more related to their local practices for prevention of malaria. Suggested to change the sub-title “Practices on malaria preventive measures”, instead of “Preventing MiP”. | We have modified the sub-title to “Practices for preventing MiP” on Page 12 line 187 |
| 15. Line 204-222: the title of the description is “Access to and utilization of IPT”, but the paragraph is mixed information of about IPT and other local traditional medicine or mis-believe practices as the pre-medication of malaria prevention. Please keep the description about access to and utilization of IPT, and separately mention in the next paragraph for “other pre-medication practices”. Thus, please re-arrange the description and quotations for line 204-233. | We have revised the paragraph on Page 13 lines 225-229 to exclude local or traditional practices as our focus is on ITN use and uptake of IPTp |
| 16. “Access to and utilization of ITN” and “Access to and utilization of IPT” should be first level title, not the second level sub-title under the “Malaria in pregnancy”. | Under PloS one guidelines, Results are level 1, and Malaria in Pregnancy is the second level as such “Access to and utilization of ITN” and “Access to and utilization of IPT” are level 3 sub-titles. |
| 17. Line 224: Overall, I would suggest the title as “Reasons for poor IPT uptake and less use of ITN” instead of “Factors influencing IPT uptake and use of ITN”. This session is mixed different findings and better to have separate sub-titles. Edit the English language in the descriptions and reduce the writing to be more concrete and scientific writing. | Thank you for your kind suggestion concerning the title. We, however, believe the current title better reflects the message of the study better. The section is mixed as it presents data from varied sources (pregnant women, caregivers, health workers, TBAs etc) in a bid to reflect the various factors at the individual, social and health sector levels that influence access and uptake of IPTp and ITNs. It, therefore, presents a mix of experiences and reflections from various levels which at times, cut across the various socio-ecological framework. We have further edited and reduced our sentence length for conciseness. |
| 18.   Please re-organize the findings (descriptions and quotations) with the following sub-titles.  a.Fear of side-effect (line 235-239)  b.Lack of knowledge and poor health education (line 249-257)  What do you mean by “sensitisation” in line 249? Please clarify or use another suitable terminology.  c.Financial barriers (line 267-276)  d.Attitude of health care providers (line 290-299)  e.Influence of decision makers (line 315-323)  f.Public health facilities versus TBA or faith-based clinics (line 338-349) | We have reorganised sub-titles for the findings as suggested  We use the word “sensitization” as a creation of awareness, gaining knowledge or health education. We have revised to “health education/awareness” |
| 19. Discussion should be re-written for more comprehensiveness, point by point. You may want to describe as “First”, “Second”, “Third” points, etc. And discussion should lead to the recommendations for the public health importance and policy input. | Thank you for your suggestions. We have further shortened our sentences for clarity in the discussion. We do not describe in that way suggested because our findings are in no way necessarily ordered as we consider all points in the discussion equally relevant. Also, because we use three levels of the socio-ecological framework (in our manuscript) ie individual, social and health system, it is important to show the findings that cut across or between these levels, which could hinder or potentially enable uptake of these interventions. |
| 20. The term “in line with the literature” should not be used and please elaborate what are the similar findings or difference in findings from other literatures. | We have rephrased for clarity on page 21 line 394 |
| 21. Please cite more references for the findings and recommendation in the discussion session. | Thank you for your suggestions. We provided a recommendation and references for every paragraph of the discussion. Our recommendations reflect our contextual interpretation of how the findings could be harnessed to improve coverage and uptake of IPTp and ITNs. We also cite 16 references in our discussion section to back up our study findings. For brevity, we have selected the most relevant and pertinent references. |
| Please make sure the point in the discussion must be consistent with and mentioned in the findings. Eg. findings about drug stock out was not clearly mentioned in the results session, but the discussion talked about the drug stock out for a paragraph. Please re-check. | Thank you for your comment. We have all gone through the entire manuscript again and confirm that the points in the discussion are all consistent with the study results. Drug stock-outs are mentioned under the sub-title “Financial barriers” (Page 16 lines 283 to 302). Financial barriers prevent pregnant women from accessing the interventions, which are expected to be freely provided at public health facilities, but due to stock-outs of IPTp and ITNs at these public health facilities, health providers write out prescriptions for purchase of IPTp and ITNs from commercial sources. Pregnant women mainly in rural and lower-socio economic communities have to purchase IPTp and/or ITNs out of pocket, contributing further to their impoverishment. |
| Conclusions should be linked to the discussion and focus on the recommendation and policy implication from the study findings. | Thank you for your suggestion. We strongly believe that all our recommendations and conclusions are related to the results and discussion. In our discussion, we focus on a) poor knowledge of IPTP and use of ITN at the individual and social levels which is why we recommend health literacy programs to address this poor awareness; b) we elaborate on choice and utilisation of ANC providers and how that influences access to IPTp and ITNs and recommend that the public health sector should work hand in hand with the private sector ie TBAs and faith-based maternity homes as they provide opportunities for leveraging on their acceptability to improve IPTp and ITN uptake; and c) we indicate that as a cross-cutting factor, health literacy programs for IPTp and ITNs should expand to include families and community members since the results and discussions show they have a strong influence on pregnant women’s choice of ANC providers, which in turn influences access to IPTp and ITNs. |
| Reviewer #2 | |
| Abstract:Line 2: Insert ‘in’ between particularly and Africa. | Included as suggested on Page 4 line 47 |
| Table 1: row 1; column 4 “No participants” using ‘No’ to represent number, may be misleading. | Clarified as suggested. Table 1 has been revised on Page 9 |
| Provide a description of the study site/area. | Included as suggested on pages 6 and 7, lines 106-126 |
